# The relationship between baseline diastolic dysfunction and postimplantation invasive hemodynamics with transcatheter aortic valve replacement

**DOI:** 10.1002/clc.23457

**Published:** 2020-09-22

**Authors:** Anthony A. Bavry, Taishi Okuno, Seyed Hossein Aalaei‐Andabili, Dharam J. Kumbhani, Stefan Stortecky, Masahiko Asami, Jonas Lanz, Stephan Windecker, Thomas Pilgrim

**Affiliations:** ^1^ Department of Medicine University of Texas Southwestern Dallas Texas USA; ^2^ Department of Cardiology, Inselspital, Bern University Hospital University of Bern Bern Switzerland; ^3^ Department of Medicine University of Maryland Baltimore Maryland USA; ^4^ Division of Cardiology Mitsui Memorial Hospital Tokyo Japan

**Keywords:** aortic stenosis, aorto‐ventricular index, diastolic dysfunction, filling pressures, invasive hemodynamics, prognosis, transcatheter aortic valve replacement

## Abstract

**Background:**

Abnormal invasive hemodynamics after transcatheter aortic valve replacement (TAVR) is associated with poor survival; however, the mechanism is unknown.

**Hypothesis:**

Diastolic dysfunction will modify the association between invasive hemodynamics postTAVR and mortality.

**Methods:**

Patients with echocardiographic assessment of diastolic function and postTAVR invasive hemodynamic assessment were eligible for the present analysis. Diastology was classified as normal or abnormal (Stages 1 to 3). The aorto‐ventricular index (AVi) was calculated as the difference between the aortic diastolic and the left ventricular end‐diastolic pressure divided by the heart rate. AVi was categorized as abnormal (AVi < 0.5 mmHg/beats per minute) or normal (≥ 0.5 mmHg/beats per minute).

**Results:**

From 1339 TAVR patients, 390 were included in the final analysis. The mean follow‐up was 3.3 ± 1.7 years. Diastolic dysfunction was present in 70.9% of the abnormal vs 55.1% of the normal AVi group (*P* < .001). All‐cause mortality was 46% in the abnormal vs 31% in the normal AVi group (*P* < .001). Adjusted hazard ratio (HR) for AVi < 0.5 mmHg/beats per minute vs AVi ≥0.5 mmHg/beats per minute for intermediate‐term mortality was (HR = 1.5, 95% confidence interval [CI] 1.1 to 2.1, *P* = .017). This association was the same among those with normal diastolic function and those with diastolic dysfunction (*P* for interaction = .35).

**Conclusion:**

Diastolic dysfunction is prevalent among TAVR patients. Low AVi is an independent predictor for poor intermediate‐term survival, irrespective of co‐morbid diastolic dysfunction.

**Abbreviations**
AVi: aorto‐ventricular index
CI: confidence interval
HR: hazard ratio
TAVR: transcatheter aortic valve replacement

## 1 INTRODUCTION

Hemodynamic assessment was initially developed as a supplemental tool to assess for paravalvular aortic regurgitation after transcatheter aortic valve replacement (TAVR).^1^, ^2^ In the current era, which is characterized by a low prevalence of paravalvular aortic regurgitation,^3^, ^4^ abnormal hemodynamics has been associated with excess intermediate‐term mortality.^5^ The aorto‐ventricular index (AVi) simultaneously examines the aortic diastolic pressure, left ventricular end‐diastolic pressure, and heart rate. An abnormal AVi (ie, < 0.5 mmHg/beats per minute) has been associated with an increased hazard for intermediate‐term mortality, independent of significant paravalvular aortic regurgitation and other confounding variables; however, the role of diastolic dysfunction is unclear. It is possible that the harmful association of abnormal AVi could be modified after controlling for baseline diastolic dysfunction. Our hypothesis is that diastolic dysfunction will modify the association between invasive hemodynamics postTAVR and late mortality.

## 2 MATERIALS AND METHODS

### 2.1 Patient population

All patients undergoing TAVR at the University of Bern were consecutively enrolled in an institutional database that is part of the Swiss TAVI Registry (clinicaltrials.gov, NCT01368250).^6^ For this analysis we required that patients had a pre‐operative echocardiogram that was evaluable for diastolic dysfunction and invasive hemodynamic assessment was performed after TAVR. Patients with a history of atrial fibrillation, previous permanent pacemaker implantation, mitral annular calcification, previous mitral valve replacement, and moderate to severe paravalvular aortic regurgitation were excluded.

The local ethics committee approved this study. All study participants gave written informed consent. Baseline variables and clinical follow‐up data were collected and recorded prospectively. Diastolic dysfunction data was re‐evaluated in the Core lab by reviewers blinded to clinical outcomes and retrospectively entered in the database. Data was entered in an online database held at the Clinical Trial Unit at the University of Bern, Switzerland.

### 2.2 Assessment of left ventricular diastolic function

All patients underwent a transthoracic echocardiogram within 3 months before TAVR. During the echocardiogram, at least three consecutive heart beats were recorded and averaged for each parameter. Echocardiogram loop and still frames were analyzed at a workstation for offline analysis (Syngo Dynamics Workplace, version 9.5, Siemens Medical Solutions, Inc., Malvern, Pennsylvania). According to current American Society of Echocardiography and European Association of Cardiovascular Imaging guidelines, the following variables were required for assessment of diastolic dysfunction (see flow diagram from previous publication): (a) annular e' velocity (septal e' < 7 cm/s or lateral e' < 10 cm/s); (b) average E/e' ratio > 14; (c) left atrial maximum volume index >34 mL/m^2^; and (d) peak tricuspid regurgitation velocity > 2.8 m/s.^7^ See flow diagram from previous publication for detailed description of assessment and gradient of diastolic dysfunction.^8^ Left ventricular diastolic dysfunction was present if three or more of the parameters were abnormal. In patients with left ventricular diastolic dysfunction, the variables for categorizing severity of left ventricular diastolic dysfunction were mitral flow velocities (E/A ratio and peak E velocity). Grade 1 was defined as an E/A ratio ≤ 0.8 with a peak E velocity of ≤0.5 m/s. Grade 3 was defined as an E/A ratio ≥ 2, with an elevation of the mean left atrial pressure. When the E/A ratio was ≤0.8 and the peak E velocity was >0.5 m/s, or if the E/A ratio was >0.8 to <2, the following were considered: (a) peak continuous‐wave Doppler velocity of the tricuspid regurgitation jet obtained from multiple views; (b) E/e' ratio; and (c) maximal left atrial volume index. If two or more of the available parameters were abnormal, diastolic dysfunction was categorized as Grade 2. If zero or one of the parameters were abnormal, Grade 1 diastolic dysfunction was present. If only one parameter was available, the diastolic dysfunction grade could not be reported. Diastolic dysfunction was also not reported if there was a discrepancy between the two available parameters. However, if neither of the two available parameters were abnormal, diastolic dysfunction was categorized as Grade 1, whereas Grade 2 diastolic dysfunction was present if both parameters were abnormal. Patients with normal diastolic function were classified as Stage 0.

### 2.3 Procedure and assessment of invasive hemodynamics

A multidisciplinary heart team of cardiac surgeons, interventional cardiologists, imaging, and heart failure specialists decided on treatment strategy and suitability for TAVR, which was performed according to current guidelines and standard approaches.^9^, ^10^ Five to 10 minutes after valve implant, a single lumen pigtail catheter was advanced into the left ventricle, while a second single lumen pigtail catheter was placed in the ascending aorta. Both catheters were flushed and zeroed. During stable rhythm and at end‐expiration, aortic pressure, left‐ventricular pressure, and heart rate were recorded. AVi was defined as the aortic diastolic pressure minus the left‐ventricular end‐diastolic pressure divided by heart rate. AVi was categorized as <0.5 mmHg/beats per minute (ie, abnormal hemodynamics) and ≥ 0.5 mmHg/beats per minute (ie, normal hemodynamics).^5^ Total arterial compliance was defined as stroke volume index divided by aortic pulse pressure.^11^ The stroke volume index was obtained from the pre‐operative echocardiogram, while the aortic pulse pressure was obtained by invasive pressures after valve implantation.

After the intervention, all patients were monitored for at least 48 hours. Laboratory examination and a 12‐lead electrocardiogram were routinely performed immediately after the procedure and daily thereafter. In all patients, echocardiogram was performed before discharge.

### 2.4 Clinical follow‐up and endpoint assessment

Standardized clinical follow‐up was performed at 30 days, 1 year, and 5 years after TAVR. Telephone interviews, documentation from referring physicians, and hospital discharge summaries were used to ascertain clinical endpoints. All suspected adverse events were independently adjudicated according to the criteria by the valve academic research consortium‐2.^12^ The primary endpoint was all‐cause mortality up to 5 years after TAVR. Secondary endpoints included cardiovascular death, and major adverse cardiac and cerebrovascular events (MACCE). MACCE was defined as all‐cause mortality, myocardial infarction, or disabling stroke.

### 2.5 Statistical analysis

Continuous data are reported as mean ± SD and categorical variables as the number of patients and percentage. *P* values were derived from student's *t* tests for continuous and chi‐square tests for categorical variables comparing the two groups. The cumulative incidence of the primary and secondary endpoints was estimated by the Kaplan‐Meier method. Patients were censored at the event of interest, time of last contact, or the maximal follow‐up time. Cox regression was used to compare time‐to‐event data between groups. Crude hazard ratios (HR) were generated with (95% confidence intervals [CIs]) with *P* values from Wald chi‐square tests. Adjusted HR (95% CI) included all variables with a *P* value <.10 from the univariable analysis into the multivariable model.

All analyses were performed with Stata version 14.2 (StataCorp, College Station, Texas). Two‐sided *P* values <.05 were considered statistically significant.

## 3 RESULTS

Between August 2007 and December 2015, 1339 patients were enrolled in the TAVI registry. Seven hundred and seventy underwent detailed echocardiographic assessment and after excluding an additional 387 patients due to inability to grade diastolic dysfunction, lack of postTAVR invasive assessment, or moderate to severe paravalvular aortic regurgitation, 390 were included in the final cohort (Figure S1). The mean follow‐up was 3.3 ± 1.7 years.

One hundred and eighty‐nine patients (48.5%) were categorized as abnormal (AVi < 0.5) and 201 patients (51.5%) as normal (AVi > 0.5). There were no differences in baseline characteristics, except that the abnormal hemodynamics group had a higher prevalence of peripheral vascular disease and higher estimated surgical risk (Table 1). Transfemoral access was used in 99% overall, while conscious sedation was used in 77.2% of the abnormal vs 92.0% of the normal group (*P* < .001, Table 2). The overall mean AVi was 0.52 ± 0.2 mmHg/beats per minute; 0.34 ± 0.2 mmHg/beats per minute in the abnormal hemodynamics group, and 0.64 ± 0.2 mmHg/beats per minute in the normal hemodynamics group (*P* < .001). Mean total arterial compliance was the same in both groups. Intra‐procedural hemodynamic variables are provided in Table 2.

**TABLE 1:** Baseline characteristics according to aorto‐ventricular index category

Variable, n (%)	AVi < 0.5 (n = 189)	AVi ≥0.5 (n = 201)	Total (n = 390)	*P* value
Age, mean years	81.6 ± 6.3	81.9 ± 5.8	81.7 ± 6.1	.58
Male sex	89 (47.1)	104 (51.7)	193 (49.5)	.36
BMI, mean kg/m^2^	26.4 ± 4.9	26.4 ± 5.3	26.4 ± 5.1	.99
Diabetes mellitus	53 (28.0)	44 (21.9)	97 (24.9)	.20
Hypercholesterolemia	120 (63.5)	124 (61.7)	244 (62.6)	.75
Hypertension	162 (85.7)	167 (83.1)	329 (84.4)	.49
Previous MI	37 (19.6)	26 (12.9)	63 (16.1)	.10
Previous PCI	58 (30.7)	49 (24.4)	107 (27.4)	.17
Previous CABG	29/184 (15.8)	19/196 (9.7)	48/380 (12.6)	.09
Previous Stroke/TIA	17 (9.0)	18 (8.9)	35 (9.0)	1.00
PVD	26 (13.8)	14 (7.0)	40 (10.2)	.03
COPD	23 (12.2)	23 (11.4)	46 (11.8)	.88
Renal insufficiency^a^	135 (71.4)	143 (71.1)	278 (71.3)	1.00
Logistic Euro score, mean	22.2 ± 15.1	16.6 ± 11.1	19.4 ± 13.5	<.001
STS score, mean	6.5 ± 4.2	4.9 ± 2.8	5.7 ± 3.6	<.001

*Note*: AVi, aortoventricular index, reported as mmHg/beats per minute.

Abbreviations: AVi, aortoventricular index, BMI, body mass index, CABG, coronary artery bypass grafting, COPD, chronic obstructive pulmonary disease, MI, myocardial infarction, PCI, percutaneous coronary intervention, PVD, peripheral vascular disease, TIA, transient ischemic attack.

^a^ Defined as estimated glomerular filtration rate < 60 mL/minutes/m^2^.

**TABLE 2:** Procedural characteristics according to aorto‐ventricular index category

Variable, n (%)	AVi < 0.5	AVi ≥0.5	Total	*P* value
Conscious sedation	146 (77.2)	185 (92.0)	331 (84.9)	<.001
Transfemoral access	187 (98.9)	199 (99.0)	386 (99.0)	.13
Valve type:				
CoreValve	75 (39.7)	74 (37.2)	149 (38.2)	.25
Sapien 3	45 (23.8)	62 (30.8)	107 (27.4)	
Sapien XT	35 (18.5)	39 (19.6)	74 (19.0)	
Evolut R	14 (7.4)	7 (3.5)	21 (5.4)	
Lotus	13 (6.9)	17 (8.5)	30 (7.7)	
Other valves	7 (3.7)	2 (1.0)	9 (2.3)	
Postimplant hemodynamics:				
Aortic systolic blood pressure, mmHg	129.4 ± 28.2	148.7 ± 27.4	139.3 ± 29.4	<.001
Aortic diastolic blood pressure, mmHg	50.1 ± 11.0	66.7 ± 14.1	58.6 ± 15.1	<.001
LVEDP, mmHg	25.5 ± 11.1	21.3 ± 8.2	23.3 ± 9.9	<.001
Heart rate, beats per minute	73.1 ± 16.4	67.4 ± 12.5	70.1 ± 14.8	<.001
AVi, mmHg/beat per minute	0.34 ± 0.2	0.68 ± 0.2	0.52 ± 0.2	<.001
Total arterial compliance	0.45 ± 0.2	0.45 ± 0.2	0.45 ± 0.2	.89

*Note*: AVi, aortoventricular index, reported as mmHg/beats per minute.

Abbreviations: AVi, aorto‐ventricular index; LVEDP, left‐ventricular end‐diastolic pressure.

Diastolic dysfunction was present in 70.9% of the abnormal AVi group vs 55.1% of the normal AVi group (*P* < .001) (Table 3). Diastolic dysfunction Stage 3 was present in 20.6% of the abnormal AVi group vs 11.4% of the normal AVi group (*P* = .018).

**TABLE 3:** Preoperative echocardiography data according to aorto‐ventricular index category

Variable	AVi <0.5	AVi ≥0.5	Total	*P* value
Left ventricular systolic function				
Left ventricular ejection fraction, %	53.0 ± 16.1 (n = 178)	56.2 ± 13.5 (n = 192)	54.6 ± 14.8 (n = 370)	.037
Stroke volume index, cc/beat/m^2^	31.8 ± 11.6 (n = 146)	34.2 ± 13.2 (n = 147)	33.0 ± 12.5 (n = 293)	.09
Left ventricular diastolic function				
E/A ratio	1.5 ± 1.1 (n = 183)	1.3 ± 1.0 (n = 197)	1.4 ± 1.1 (n = 380)	.075
E wave, m/sec	0.95 ± 0.40 (n = 182)	0.80 ± 0.31 (n = 197)	0.90 ± 0.36 (n = 379)	<.001
A wave, m/sec	0.87 ± 0.42 (n = 182)	0.82 ± 0.34 (n = 197)	0.84 ± 0.38 (n = 379)	.21
E/e' ratio	23.6 ± 11.9 (102)	19.7 ± 12.6 (n = 123)	21.5 ± 12.4 (n = 225)	.02
e', cm/sec	4.5 ± 1.3 (n = 101)	4.6 ± 1.6 (n = 121)	4.5 ± 1.5 (n = 222)	.74
Deceleration time, msec	223.6 ± 85.0 (n = 181)	241.6 ± 86.6 (n = 196)	232.9 ± 86.2 (n = 377)	.043
Isovolumic relaxation time, msec	78.2 ± 23.5 (n = 177)	81.1 ± 22.6 (n = 194)	79.7 ± 23 (n = 371)	.23
Tricuspid regurgitation velocity, m/sec	2.9 ± 0.6 (n = 129)	2.8 ± 0.5 (n = 121)	2.8 ± 0.5 (n = 250)	.08
Left atrial volume index, mL/m^2^	44.6 ± 17.1 (n = 183)	41.3 ± 17.1 (n = 198)	42.9 ± 17.1 (n = 381)	.065
Diastolic dysfunction Stage:				
0	55 (29.1%)	90 (44.8%)	145 (37.2%)	.002
1	26 (13.8%)	27 (13.4%)	53 (13.6%)	1.00
2	69 (36.5%)	61 (30.3%)	130 (33.3%)	.20
3	39 (20.6%)	23 (11.4%)	62 (15.9%)	.018
Valvular regurgitation				
Aortic regurgitation ≥ moderate	4/70 (5.7%)	4/58 (7.0%)	8/128 (6.2%)	1.0
Mitral regurgitation ≥ moderate	32/187 (17.1%)	24/198 (12.1%)	56/385 (14.5%)	.19
Tricuspid regurgitation ≥ moderate	27/187 (14.4%)	14/198 (7.1%)	41/385 (10.6%)	.02
Aortic stenosis severity				
Aortic valve area, cm^2^	0.76 ± 0.20 (n = 70)	0.76 ± 0.30 (n = 59)	0.76 ± 0.26 (n = 129)	.97
Mean aortic valve gradient, mmHg	41.5 ± 20.4 (n = 167)	43.2 ± 17.3 (n = 173)	42.3 ± 18.9 (n = 340)	.41
Left ventricular hypertrophy				
Relative wall thickness	0.53 ± 0.2 (n = 160)	0.54 ± 0.2 (n = 169)	0.53 ± 0.5 (n = 329)	.60
LV mass index, g/m^2^	144.2 ± 52.7 (n = 162)	140.0 ± 51.7 (n = 175)	142 ± 52.2 (n = 337)	.47

*Note*: AVi, aortoventricular index, reported as mmHg/beats per minute.

Abbreviations: AVi, aorto‐ventricular index; EF, ejection fraction; LV, left ventricular.

All‐cause mortality was 46% in the abnormal AVi group vs 31% in the normal AVi group (*P* = .001) (Figure S2). After multivariable adjustment, AVi < 0.5 was an independent predictor for intermediate‐term mortality (HR = 1.5, 95% CI 1.1 to 2.1; *P* = .017, Table 4). Complete outcomes are provided in Table S1.

**TABLE 4:** Univariable and multivariable analysis examining aorto‐ventricular index as a function of intermediate‐term mortality

Univariable analysis	HR	95% CI	*P* value	Multivariable analysis	HR	95% CI	*P* value
AVi < 0.5	1.9	1.2‐2.8	.003	AVi < 0.5	1.5	1.1‐2.2	.017
Diastolic dysfunction	3.2	2.0‐5.1	<.001	Diastolic dysfunction	1.7	1.1‐2.5	.011
PAD	3.5	1.7‐6.9	<.001	PAD	1.8	1.2‐28	.008
MR ≥ moderate	2.7	1.5‐4.8	.001	MR ≥ moderate	1.8	1.2‐2.7	.006
COPD	2.6	1.4‐4.9	.003	COPD	2.0	1.3‐3.1	.001
Male gender	1.5	1.01‐2.3	.048				
STS risk score	1.06	1.02‐1.1	.002				
Mean aortic gradient ≥40 mmHg	1.5	0.94‐2.3	.095				
TR ≥moderate	2.5	1.3‐4.9	.006				
NYHA class III/IV	1.7	1.1‐2.6	.029				
Ejection fraction <40%	3.8	1.7‐5.2	<.001				
Diabetes mellitus	1.7	1.1‐2.7	.03				

*Note*: AVi, aortoventricular index, reported as mmHg/beats per minute.

Abbreviations: AVi, aortoventricular index; COPD, chronic obstructive pulmonary disease; MR, mitral regurgitation; NYHA, New York heart association; PAD, peripheral arterial disease; TR, tricuspid regurgitation.

**FIGURE 1:**
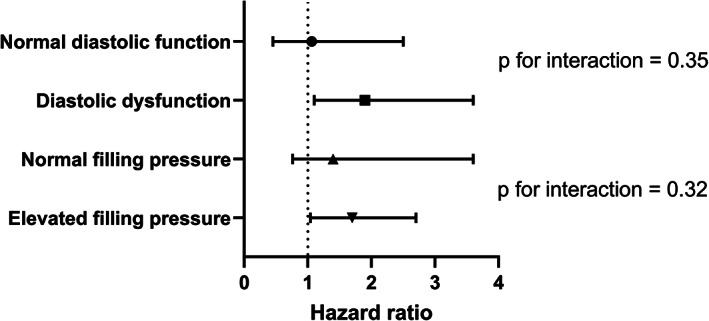
Association of low vs normal AVi on mortality according to subgroups. Hazard ratio > 1.0 indicates that low AVi is associated with increased mortality compared with normal AVi. Normal estimated filling pressures are defined as normal diastolic function/diastolic dysfunction Grade 1, while elevated filling pressure as diastolic dysfunction Grade 2 or 3. AVi, aorto‐ventricular index

Among those with normal diastolic function, all‐cause mortality was 27% in the AVi < 0.5 mmHg/beats per minute vs 29% in the AVi ≥0.5 mmHg/beats per minute group (*P* = .67). The multi‐variable association between low vs normal AVi was (HR = 1.06, 95% CI 0.45 to 2.50, *P* = .89). Among those with diastolic dysfunction, all‐cause mortality was 59% in the AVi < 0.5 vs 44% in the AVi ≥0.5 group (*P* = 0.006). The multi‐variable association between low vs normal AVi was (aHR = 1.90, 95% CI 1.10 to 3.60, *P* = .029; p for interaction = 0.35) (Figure 1).

We also categorized subjects according to normal estimated baseline filling pressure (normal diastolic function and diastolic dysfunction Stage 1, which was present in 50.8% and elevated estimated baseline filling pressure (diastolic dysfunction Stage 2 and 3, which was present in 49.2%. Among those with normal baseline filling pressures, the association between low vs normal AVi was (HR = 1.4, 95% CI 0.76 to 3.6, *P* = .34) and among those with elevated baseline filling pressures the association was (HR = 1.7, 95% CI 1.04 to 2.7, *P* = .033; *P* for interaction = .32) (Figure 1).

## 4 DISCUSSION

The pertinent findings from the present analysis can be summarized as follows: (a) Baseline diastolic dysfunction was prevalent among TAVR patients. (b) Postimplantation AVi < 0.5 mmHg/beats per minute was an independent predictor for poor intermediate‐term survival. (c) Abnormal invasive hemodynamics was associated with poor prognosis irrespective of baseline diastolic dysfunction. We previously theorized that each component of the AVi would have clinical significance. For example, a low aortic diastolic pressure could represent unrecognized paravalvular aortic regurgitation, poor systemic flow, and/or poor arterial compliance. An elevated left ventricular end‐diastolic pressure could represent left ventricular systolic dysfunction and/or diastolic dysfunction. An elevated heart rate could represent poor systemic flow (since heart rate is inversely related to stroke volume) and/or background medical therapy. Therefore, the AVi is more than just an assessment of diastolic dysfunction, which was born out in the study findings.

These findings confirm our previous observation of an association between abnormal hemodynamics and poor survival, but this time in a larger independent cohort. The current analysis leveraged the Bern TAVI registry, which represents the largest published work on diastology and TAVR. Diastolic dysfunction was prevalent among all patients but especially among those with abnormal hemodynamics; however, pre‐operative diastolic dysfunction did not modify the association between abnormal AVi and intermediate‐term survival. We found no evidence for a difference in total arterial compliance between groups, which was another potential mechanism for the hazardous association.

Moderate to severe paravalvular aortic regurgitation has consistently been shown to be associated with poor survival^4^, ^13^, ^14^, ^15^, ^16^, ^17^, ^18^; therefore, we excluded these patients from analysis. The prognostic significance of mild aortic regurgitation is not clear with some studies revealing no association,^4^, ^19^ and others revealing a hazardous association.^20^ Our results to not apply to patients who have moderate to severe paravalvular aortic regurgitation.

We previously reported that diastolic dysfunction Stage 3 was the strongest predictor for 1 year mortality. Optimal management of left ventricular diastolic dysfunction among TAVR patients is not well known.^21^ Diastolic dysfunction remains the same in half or more of patients 1 year after TAVR,^8^, ^22^ and myocardial fibrosis remains unchanged 9 months after surgical AVR.^23^ However, patients across the spectrum of diastolic dysfunction have been shown to improve health status as early as 1 month after TAVR.^24^ The mechanism for the low AVi and excess mortality association remains unclear. Patients with abnormal invasive hemodynamics may be more sensitive to volume overload from even mild paravalvular aortic regurgitation.^25^ It is also possible that patients with abnormal hemodynamics are particularly sensitive to heart rate, blood pressure, and filling pressures, which could be influenced by background medical therapy. It is also possible that infiltrative myocardial disease processes such as cardiac amyloidosis could be prevalent in some of the patients with abnormal hemodynamics.^26^, ^27^

Limitations of the current analysis include: (a) Abnormal hemodynamics was present in approximately one‐fifth of our derivation cohort vs approximately one‐half of patients in the current cohort. The reason for this difference is unknown. Aortic diastolic blood pressure was the same between the two cohorts with abnormal hemodynamics; however, the Bern TAVI registry was associated with a higher left ventricular end‐diastolic pressure and heart rate. This may reflect differences in background/procedural medical therapy; however, this information was not available in either registry. (b) Patients with mitral annular calcification, atrial fibrillation, previous pacemaker or surgical mitral valve were excluded from the current study. This could have resulted in unintended selection bias; however, these characteristics were included in our development cohort.^5^ (c) Residual confounding cannot be excluded due to the observational nature, relatively modest size of the database, and inability to perform propensity analysis. (d) It is possible that poor prognosis from abnormal hemodynamics was influenced by mild paravalvular aortic regurgitation. Although we excluded patients with moderate to severe paravalvular aortic regurgitation, we were unable to distinguish no/trivial/mild paravalvular aortic regurgitation among the analyzed cohort. However, in our development cohort, we excluded patients with mild paravalvular aortic regurgitation and still noted a harmful association from abnormal hemodynamics.^5^ (e) Total arterial compliance was calculated from stroke volume index which was derived from pre‐operative echocardiography, while pulse pressure was obtained from intra‐operative invasive pressures. (f) We were unable to examine sub‐groups with low ejection fraction/low‐gradient aortic stenosis.

## 5 CONCLUSION

In summary, invasive hemodynamic assessment after TAVR can be used to risk stratify patients. There was a high prevalence of diastolic dysfunction among TAVR patients. AVi < 0.5 mmHg/beats per minute was associated with poor intermediate‐term survival, and baseline diastolic dysfunction did not modify this association.

## CONFLICT OF INTEREST

Dr Anthony Bavry has received honoraria from the American College of Cardiology and Edwards Lifesciences (significant); Dr Taishi Okuna has receive lecture fees from Abbott (modest); Dr Stephan Windecker received research and educational grants to the institution from Abbott, Amgen, Bayer, BMS, Biotronik, Boston Scientific, CSL Behring, Edwards, Medtronic, Polares and Sinomed; Dr Thomas Pilgram received research grants to the institution from Biotronik, Boston Scientific, Edwards Lifesciences; speaker fees from Biotronik and Boston Scientific; consultancy for HighLifeSAS.

## Supporting information


**Figure S1** Flow diagram of study cohort

**Figure S2** Survival analysis among subjects with low vs normal Aorto‐Ventricular index (AVi)

**Table S1** Clinical outcomes

## Data Availability

The data that support the findings of this study are available from the corresponding author upon reasonable request.
