# Supplementary material for: The relationship between baseline diastolic dysfunction and postimplantation invasive hemodynamics with transcatheter aortic valve replacement
Source: Clin Cardiol. 2020 Sep 22;43(12):1428–34. doi: 10.1002/clc.23457 (PMC7724241; doi:10.1002/clc.23457)
Supplement: Supplementary file 1 — Figure S1 Flow diagram of study cohort [file CLC-43-1428-s001.pdf]

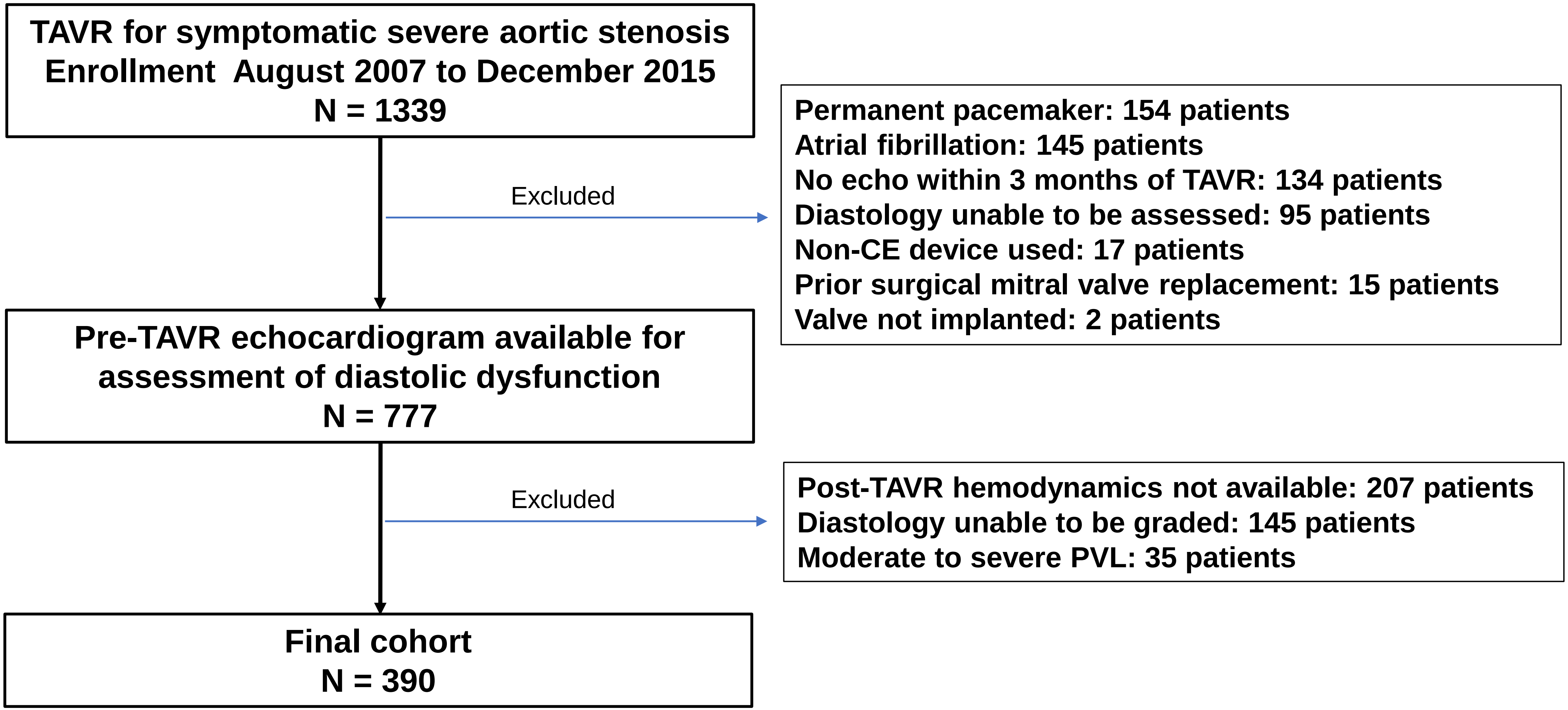
TAVR for symptomatic severe aortic stenosis
Enrollment August 2007 to December 2015
N = 1339
Excluded
Permanent pacemaker: 154 patients
Atrial fibrillation: 145 patients
No echo within 3 months of TAVR: 134 patients
Diastology unable to be assessed: 95 patients
Non-CE device used: 17 patients
Prior surgical mitral valve replacement: 15 patients
Valve not implanted: 2 patients
Pre-TAVR echocardiogram available for assessment of diastolic dysfunction
N = 777
Excluded
Post-TAVR hemodynamics not available: 207 patients
Diastology unable to be graded: 145 patients
Moderate to severe PVL: 35 patients
Final cohort
N = 390
