# Supplementary figures and images for: The relationship between baseline diastolic dysfunction and postimplantation invasive hemodynamics with transcatheter aortic valve replacement
Source: Clin Cardiol. 2020 Sep 22;43(12):1428–34. doi: 10.1002/clc.23457 (PMC7724241; doi:10.1002/clc.23457)

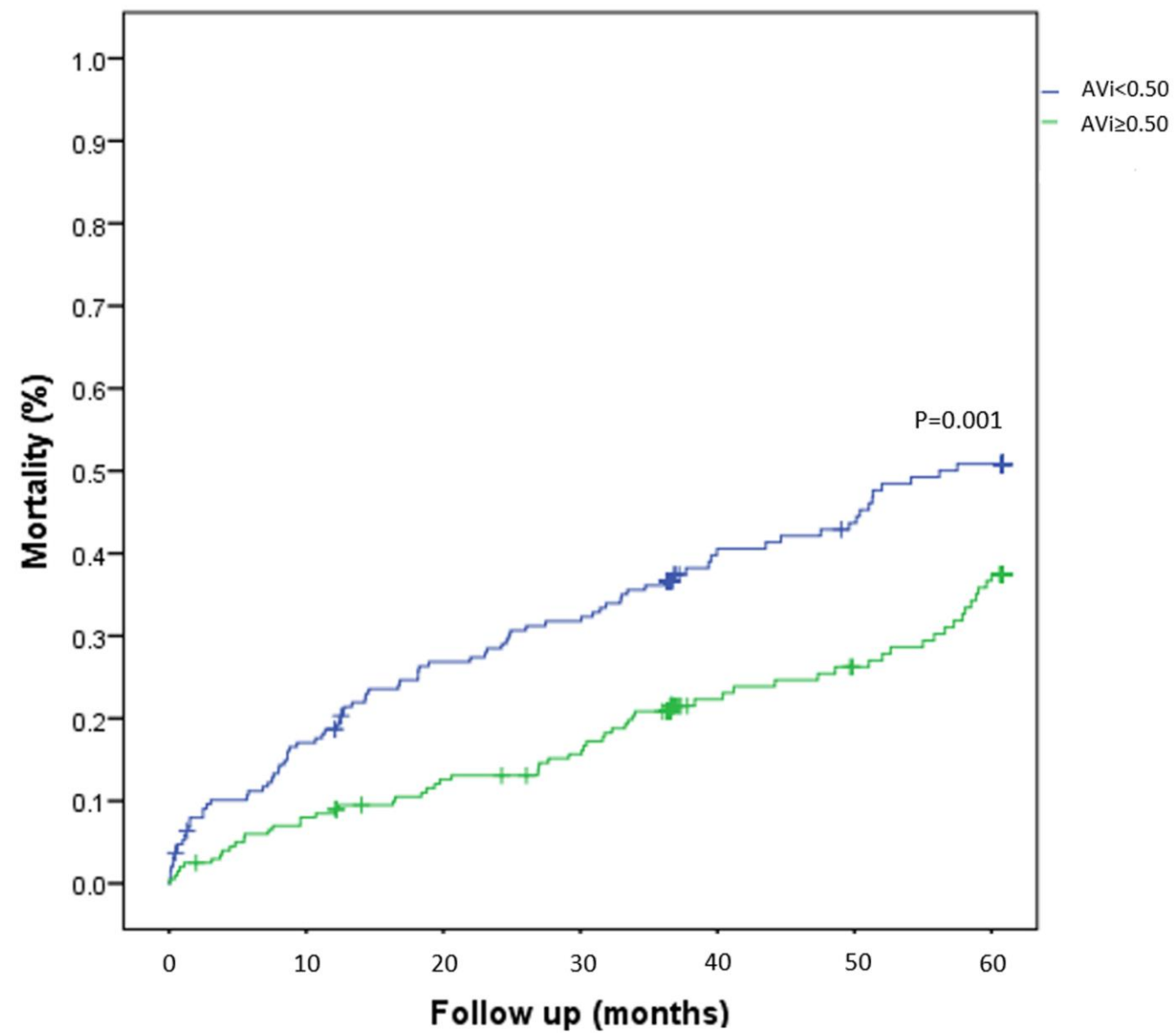

|                          |     |     |     |     |    |    |    |
|--------------------------|-----|-----|-----|-----|----|----|----|
| Number at risk, AVi<0.50 | 189 | 155 | 134 | 125 | 76 | 71 | 62 |
| Number at risk, AVi≥0.50 | 201 | 184 | 171 | 163 | 99 | 92 | 79 |

Supplement: Supplementary file 2 — Figure S2 Survival analysis among subjects with low vs normal Aorto‐Ventricular index (AVi) [file CLC-43-1428-s002.pdf]
