# Supplementary material for: The relationship between baseline diastolic dysfunction and postimplantation invasive hemodynamics with transcatheter aortic valve replacement
Source: Clin Cardiol. 2020 Sep 22;43(12):1428–34. doi: 10.1002/clc.23457 (PMC7724241; doi:10.1002/clc.23457)
Supplement: Supplementary file 3 — Table S1 Clinical outcomes [file CLC-43-1428-s003.docx]

Supplemental Table 1. Clinical outcomes

| Event; number (%) | AVi < 0.5 | AVi ≥ 0.5 | Total | p-value |
| --- | --- | --- | --- | --- |
| All-cause mortality, 5 yr | 86 (45.5%) | 62 (30.8%) | 148 (37.9%) | <0.001 |
| All-cause mortality, 1 yr | 35 (18.5%) | 18 (8.9%) | 53 (13.6%) | 0.005 |
| All-cause mortality, 30 d | 10 (5.3%) | 4 (2.0%) | 14 (3.6%) | 0.10 |
| CV mortality, 5 yr | 57 (30.2%) | 40 (20.0%) | 97 (24.9%) | 0.003 |
| CV mortality, 1 yr | 24 (12.7%) | 13 (6.5%) | 37 (9.5%) | 0.025 |
| CV mortality, 30 d | 9 (4.8%) | 3 (1.5%) | 12 (3.1%) | 0.08 |
| Disabling stroke, 5 yr | 8 (4.2%) | 12 (6.0%) | 20 (5.1%) | 0.64 |
| Disabling stroke, 1 yr | 5 (2.6%) | 8 (4.0%) | 13 (3.3%) | 0.56 |
| Myocardial infarction, 5 yr | 10 (5.3%) | 4 (2.0%) | 14 (3.6%) | 0.033 |
| Myocardial infarction, 1 yr | 5 (2.6%) | 2 (1.0%) | 7 (1.8%) | 0.19 |
| MACCE, 5 yr | 95 (50.3%) | 70 (34.8%) | 165 (42.3%) | <0.001 |
| MACCE, 1 yr | 38 (20%) | 25 (12.4%) | 63 (16.1%) | 0.031 |

AVi=aortoventricular index, reported as mm Hg/beats per minute; d=day; MACCE=major adverse cardiac and cerebrovascular events; yr=year
